# A novel biomimetic trabecular bone metal plate for bone repair and osseointegration

**DOI:** 10.1093/rb/rbad003

**Published:** 2023-02-06

**Authors:** Zhijie Ma, Baoyi Liu, Shuqiang Li, Xiaohu Wang, Jingyu Li, Jiahui Yang, Simiao Tian, Chengjun Wu, Dewei Zhao

**Affiliations:** Orthopaedic of Department, Affiliated ZhongShan Hospital of Dalian University, Dalian, Liaoning 116001, China; Orthopaedic of Department, Affiliated ZhongShan Hospital of Dalian University, Dalian, Liaoning 116001, China; Orthopaedic of Department, The Fourth People's Hospital of Guiyang, Guiyang, Guizhou 550000, China; Orthopaedic of Department, Affiliated ZhongShan Hospital of Dalian University, Dalian, Liaoning 116001, China; Orthopaedic of Department, Affiliated ZhongShan Hospital of Dalian University, Dalian, Liaoning 116001, China; Orthopaedic of Department, Affiliated ZhongShan Hospital of Dalian University, Dalian, Liaoning 116001, China; Orthopaedic of Department, Affiliated ZhongShan Hospital of Dalian University, Dalian, Liaoning 116001, China; School of Biomedical Engineering, Faculty of Electronic Information and Electrical Engineering, Dalian University of Technology, Dalian, Liaoning 116024, China; Orthopaedic of Department, Affiliated ZhongShan Hospital of Dalian University, Dalian, Liaoning 116001, China

**Keywords:** fracture, trabecular bone metal, bone plate, bone repair, osseointegration

## Abstract

Fracture is one of the most common traumatic diseases in clinical practice, and metal plates have always been the first choice for fracture treatment because of their high strength. However, the bone plates have high elastic modulus and do not match the biomechanics of human bone, which adversely affects callus formation and fracture healing. Moreover, the complex microenvironment in the human body can induce corrosion of metallic materials and release toxic ions, which reduces the biocompatibility of the bone plate, and may necessitate surgical removal of the implant. In this study, tantalum (Ta) was deposited on porous silicon carbide (SiC) scaffolds by chemical vapor deposition technology to prepare a novel porous tantalum (pTa) trabecular bone metal plate. The function of the novel bone plate was evaluated by implantation in an animal fracture model. The results showed that the novel bone plate was effective in fracture fixation, without breakage. Both X-ray and microcomputed tomography analysis showed indirect healing by both pTa trabecular bone metal plates and titanium (Ti) plates; however, elastic fixation and obvious callus formation were observed after fixation with pTa trabecular bone metal plates, indicating better bone repair. Histology showed that pTa promoted the formation of new bone and integrated well with the host bone. Therefore, this novel pTa trabecular bone metal plate has good prospects for application in treating fractures.

## Graphical Abstract

**Figure:**
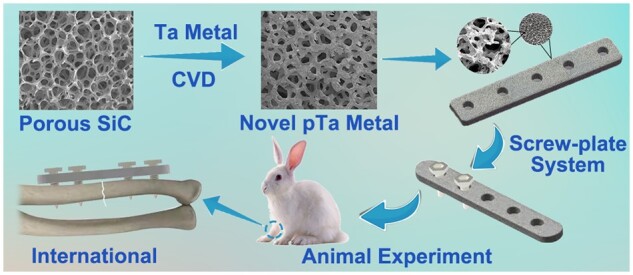

## Introduction

Fracture is the most common bone trauma in clinical practice. Mild fractures can lead to limb deformities and dysfunction and bring harm to the human body in terms of unnatural action. Severe fractures can lead to serious complications such as shock, which endangers the life of the patient if not treated quickly. The key to treating fractures is resetting and fixing the bone, thereby providing a stable environment, which plays a vital role in fracture healing. The history of the medical use of metal materials as bone plates in treating fractures traces back more than 100 years. As early as 1886, Lane first reported the application of stainless steel bone plates to treat limb fractures [1]. To date, metal materials such as stainless steel, titanium (Ti) and its alloys are still the first choice for the internal fixation of load-bearing fractures due to their advantageous strength, but the high elastic modulus of these metal materials does not match that of human bone and has adversely affect on callus formation and healing [2, 3]. In addition, metal plates might easily loosen or even release toxic ions after implantation and long-term retention in the body, which necessitates reoperation for removal [4, 5]. Therefore, these are not ideal materials for bone plates in fracture fixation.

In clinical practice, an ideal bone plate should have sufficient strength to fix fractures, an elastic modulus matching that of bone, and excellent biocompatibility and bioactivity. To reduce the elastic modulus of metallic materials, researchers have studied porous materials, which mainly include Ti fiber bone plates and 3D-printed porous Ti plates [3, 6–8]. Although the porous structure reduces the elastic modulus of the bone plate, it does not change the nature of the bone plate material [8]. In addition, polymer materials have become a flourishing of research topics, mainly including polylactic acid and magnesium-based metals [9–12]. The elastic modulus of these materials ranges from 0.5 to 10 GPa, which is similar to or even lower than that of bone tissue. Orthopedic implants made of these degradable materials do not have adverse effects such as stress shielding on bone tissue and are compatible with X-ray, magnetic resonance imaging and computed tomography (CT) examination after implantation. Medical imaging is conducive to monitoring and tracking the bone tissue at the fracture site to clearly determine the state of fracture healing [9]. Additionally, bone plates made of these degradable materials do not need to be removed by secondary surgery after implantation, which reduces the economic burden and mental stress on the patient [10]. The major drawback is that these materials are not sufficiently strong for universal application. They are more suitable for the fixation of craniofacial fractures, dental fractures and fractures at other sites with lower loads. When applied to fix fractures of bones with higher loads, these materials exhibit insufficient stiffness and poor wear resistance [11]. In addition, some degradable materials, such as magnesium-based metals, have unfavorable features, such as a high degradation rate or hydrogen formation, which limit their clinical application [12].

Tantalum (Ta) trabecular metal has attracted much more attention due to its excellent biocompatibility and bone integration performance. Trabecular bone implants made of Ta have shown excellent bone integration performance and long-term chemical stability in preclinical and clinical observations, and can thus be permanently retained in the host tissue [13–15]. However, studies on its use as a bone plate material for internal fixation system of fracture has not yet been documented. In addition, the preparation technology of Ta trabecular metal materials has been monopolized by the Zimmer Inc, with exorbitant price limiting its extensive promotion and application in the market.

In recent years, our team focused on studying the novel porous tantalum (pTa) trabecular bone metal materials and applied chemical vapor deposition (CVD) technology to prepare a novel pTa trabecular bone metal plate on porous silicon carbide (SiC) scaffolds [16–18]. Results of previous research have reported that the material made of novel pTa trabecular metal has excellent biocompatibility and biomechanical properties, and bone integration performances. Its yield strength is 45.8 ± 2.9 MPa, the pressure resistance is 61.4 ± 3.2 MPa and the elastic modulus is 4.8 GPa, which shows its potential as an internal fixation device [16]. Based on the current problems of bone plate materials, combined with the excellent biocompatibility and bone integration performance of the new bionic beams, a new type of porous metal bone panel was designed in this study. Moreover, the rabbit ulnar fracture model was used to evaluate the effect of this novel pTa trabecular bone metal plate on fracture healing and callus formation in the search for a new internal fixation system.

## Materials and methods

### Preparation of novel pTa trabecular bone metal plate

Porous SiC scaffolds coated with Ta metal were prepared by CVD to form novel pTa trabecular bone metal materials. The porous SiC scaffold was placed at one end of the reaction chamber and the Ta raw materials at the other. When the reaction chamber temperature was heated to 1050°C and the degree of vacuum dropped below 133 Pa, chlorine gas was delivered to the front end of the chlorination chamber. The chlorine gas first was reacted with the Ta raw material to form TaCl_5_, and then hydrogen gas was reacted with TaCl_5_ to form Ta, which was deposited on the porous SiC scaffold. Successfully prepared novel pTa trabecular bone metal was ultrasonically cleaned. The specific preparation method has been described previously [16–18].

**Figure 1.**
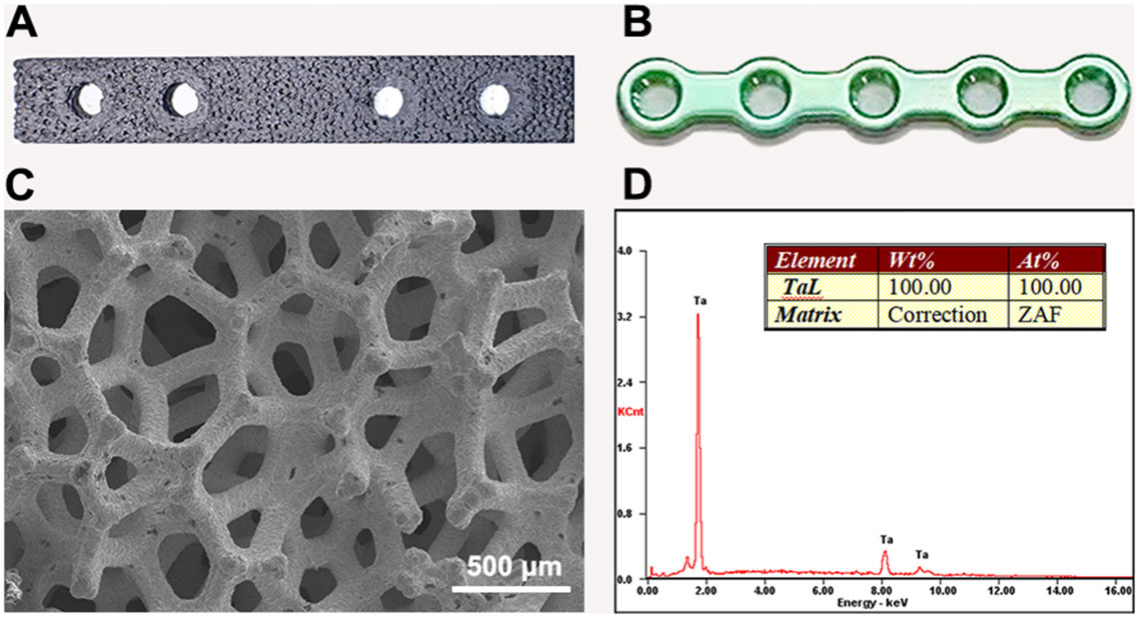
Bone plate for fracture fixation: (**A**) novel pTa trabecular metal plate; (**B**) Ti plate; (**C**) microstructure of pTa trabecular metal plate; and (**D**) EDS analysis of pTa trabecular metal plate element composition.

The overall structure of the bone plate was processed by an electrical discharge machining shaping system (EA8S, TACHIBANA, Japan). Due to the difference in mechanical strength between the bone plate with porous structure and the Ti solid plate, the thickness of the novel pTa trabecular bone metal plate was increased during designing process compared to the Ti plate. The specific dimensions are shown in Fig. 1A, where length, width, thickness and screw hole diameter are 50, 9, 2 and 2.7 mm, respectively. The miniature five-hole Ti plates and screws were purchased from Shandong Wego Orthopaedic Device Co. Ltd, China, as shown in Fig. 1B, where length, width, thickness and hole diameter are 50, 9, 1.5 and 2.7 mm, respectively.

### Animal experiment

#### Surgical procedures

**Figure 2.**
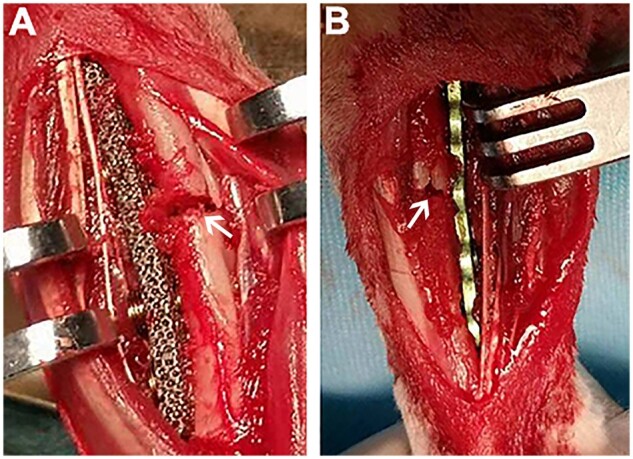
Rabbit ulnar comminuted fracture model, a 2 × 2 × 2 mm comminuted fracture was created at the center of the ulna stem. (**A**) pTa trabecular metal plate was fixed to the ulna fracture. (**B**) Ti plate was fixed to the rabbit ulna fracture. *Note*: the arrow represents the fracture gap.

Eighteen male New Zealand white rabbits weighing 2.0–2.5 kg and aged 4–6 months were randomly divided into a pTa trabecular metal plate group and a Ti plate group for the *in vivo* fracture model implantation trial, with nine rabbits in each group. After the animal was anesthetized with Lumianning (1 ml/kg), the left anterior limb hair was shaved and the limb fixed while the rabbit was on the operation table in the prone position. After disinfection, an incision approximately 5–6 cm long was made in the left anterior limb, and the subcutaneous tissue and muscle were bluntly separated to expose the middle of the ulna. A transverse fracture was modeled in the middle of the ulna using a wire saw, with a defect width of 1 mm. The fracture ends were clamped with reduction forceps and fixed with a pTa trabecular bone metal plate (Fig. 2A) or a Ti plate (Fig. 2B). Each set of fixation instrumentation consisted of one plate and four screws. After fixation and suturing, the incision was wrapped.

After the operation, the animals were kept in individual cages and resumed their activities. To prevent postoperative wound infection, 40 000 U of penicillin was intramuscularly injected into each animal on 3 consecutive days, and disinfection with iodophor was performed around the incision every day until the incision healed.

All experimental procedures were approved by and performed according to the relevant guidelines and regulations of the Ethics Committee of Affiliated Zhongshan Hospital of Dalian University (No. 2017-045).

#### X-ray evaluation of fracture healing

At 4, 8 and 12 weeks after internal fixation, the rabbit ulna was examined by X-ray imaging in the anteroposterior and lateral views by radiology technicians to evaluate the healing of the ulnar fracture. The X-ray tube was set to 70 kV and 100 mA. The development and fixation times were strictly consistent among all X-ray films.

#### Micro-CT evaluation of fracture healing

At 4, 8 or 12 weeks after internal fixation, the animals were sacrificed by the intravenous injection of air through an ear margin vein and the ulna was removed with the plate and screws. Then, the soft tissue around the bone block was completely removed and placed in the scanning cabin. Fracture healing and callus formation around the fracture were evaluated by micro-CT (Siemens, USA) performed at 80 kV and 500 μA. Radiographic data were collected and reconstructed using Inveon Acquisition Workplace (Siemens, USA) and analyzed using Inveon Research Workplace (Siemens, USA) software.

### Histological and histomorphometric evaluations

After micro-CT scanning, the obtained bone specimens were immediately fixed in 4% phosphate-buffered paraformaldehyde. The whole bone samples, with pTa trabecular bone metal plates and Ti plates, were dehydrated in a gradient of ethanol solutions (60%, 70%, 80%, 90% and 100%) and then embedded in epoxy resin. The samples were then sliced using an EXACT hard tissue slicer, and the plane that completely exposed the plate and the host bone was obtained. The thickness of the slice was 100 μm, but the slice was ground to 50–60 μm. Finally, Van Gieson staining was performed for histomorphometric analysis to evaluate the formation of new bone and the interface between the host bone and metal. The area of new bone formation was quantitatively evaluated using ImageTool v3.0 software (UTHSCSA, USA).

### Statistical analysis

SPSS 20.0 was used for statistical analysis. All values are expressed as the mean ± standard deviation. Multiple-group comparisons were performed using one-way analysis of variance (ANOVA) or two-way repeated-measures ANOVA over time. If the results indicated a statistically significant difference, the least significant difference test was used for comparisons between the two groups. *P* < 0.05 was considered statistically significant.

## Results

### General conditions of the animals

All animals were in good condition after the operation, and there was no cases of infection or exudation at the surgical wound site. After internal fixation and recovery from anesthesia, all animals could move well. No animals were undergone external fixation.

### X-ray evaluation of fracture healing and callus formation

**Figure 3.**
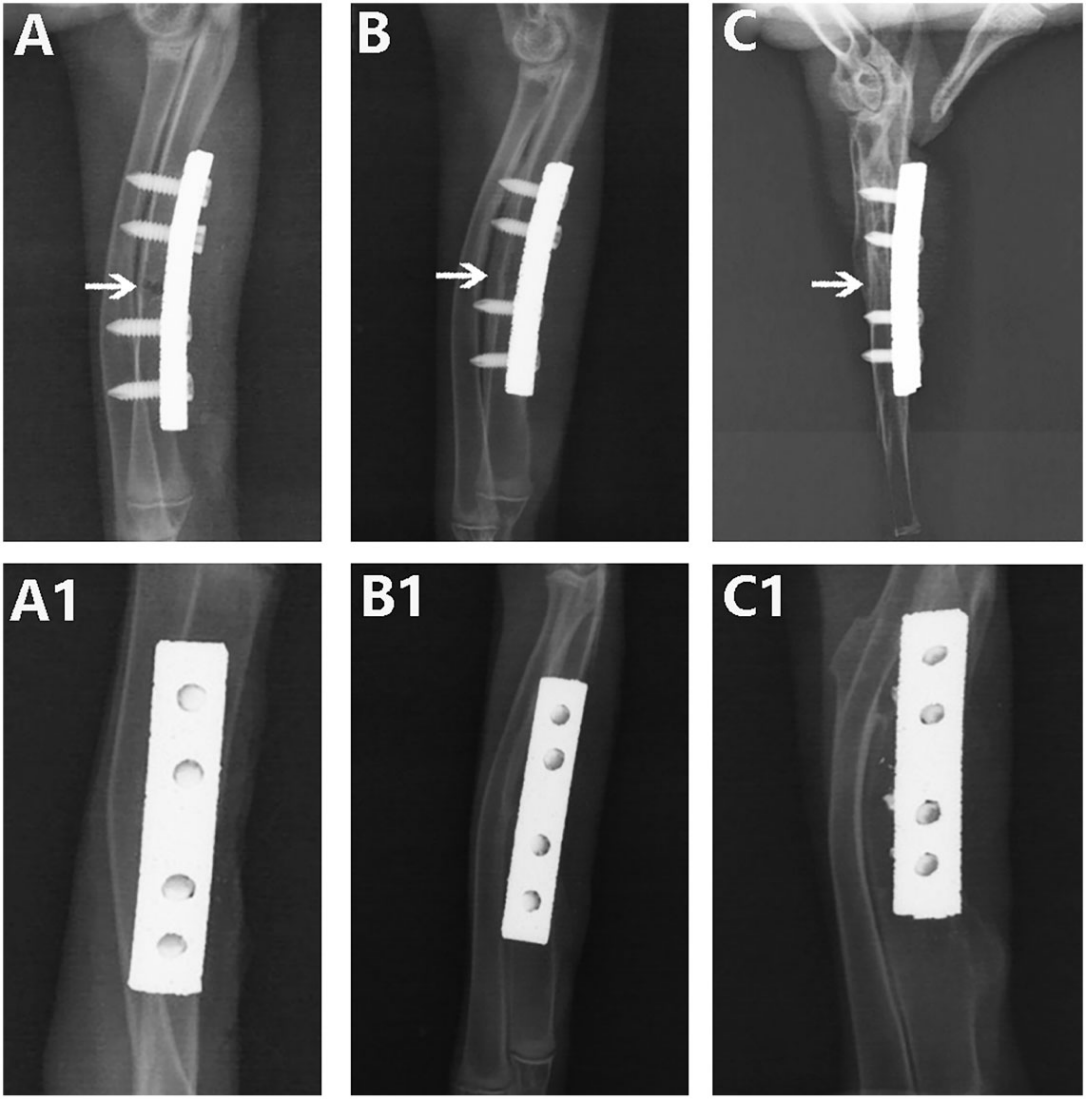
X-ray assessment of the healing state of rabbit ulna fractures with pTa trabecular bone metal plate: (**A** and **A1**) 4 weeks after the operation, anteroposterior and lateral X-ray showed callus formation around the fracture and the fracture began to healed; (**B** and **B1**) 8 weeks after the operation, anteroposterior and lateral X-ray showed a large continuous callus formation around the fracture, and the fracture was completely healed; and (**C** and **C1**) 12 weeks after the operation, anteroposterior and lateral X-ray showed that the fracture was completely healed and the medullary cavity was recanalized. *Note*: the arrow represents the fracture gap.

**Figure 4.**
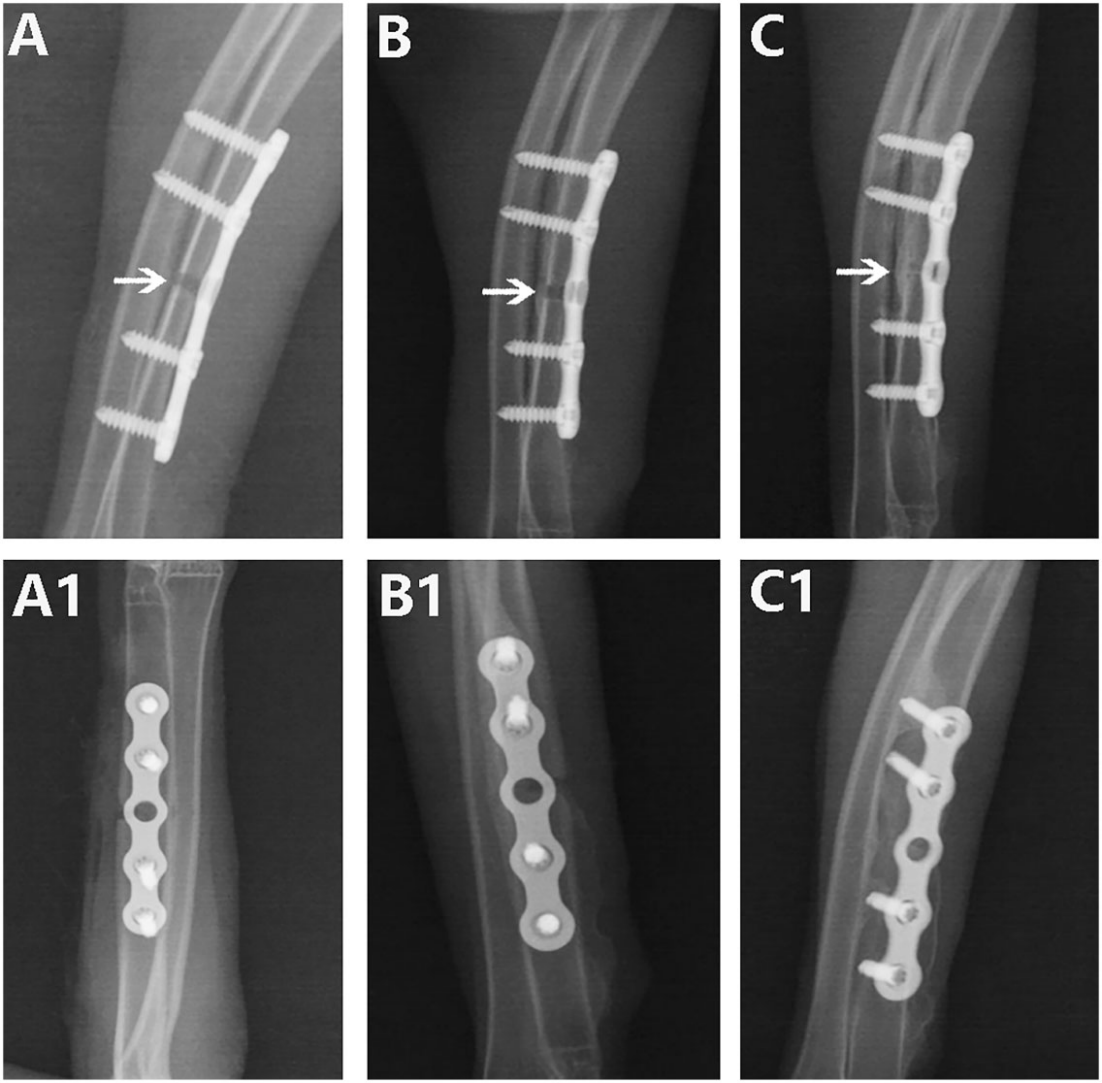
X-ray assessment of the healing state of rabbit ulna fractures with Ti plate: (**A** and **A1**) 4 weeks after the operation, anteroposterior and lateral X-ray showed a small amount of callus formation around the fracture, and the fracture began to healed; (**B** and **B1**) 8 weeks after the operation, anteroposterior and lateral X-ray showed continuous callus formation around the fracture, but the fracture line was still clear and the fracture was not completely healed; and (**C** and **C1**) surgery after 12 weeks, anteroposterior and lateral X-ray showed that the fracture healed. *Note*: the arrow represents the fracture gap.

The state fracture healing and callus formation was observed and evaluated by anteroposterior and lateral X-ray examination of the rabbit ulna. The Ti plate, pTa trabecular metal plate and screws were well fixed and not damaged by repeated X-ray observation as shown in Figs 3 and 4. During the healing process in the two groups, callus formation was observed around the fracture ends, indicating that both kinds of plates facilitated indirect healing.

According to repeated X-ray observations over 3 months, at Week 4 after the operation, continuous callus formation was observed around the fracture in both groups, indicating initial healing. However, the fracture line could be clearly observed in both groups (Figs 3A and 4A). At Week 8 after the operation, the continuous callus at the fracture ends had grown in the pTa trabecular metal plate group, and no fracture line was visible, indicating complete fracture healing (Fig. 3B). The continuous callus at the fracture ends in the Ti plate group had also grown gradually, indicating further fracture healing. However, a blurred fracture line could still be observed on anteroposterior and lateral X-ray films (Fig. 4B). At Week 12, the fractures in both groups had healed completely, but the medullary cavity showed complete reconnection only in the pTa trabecular metal plate group (Fig. 3C). In the Ti plate group, there was still sclerotic bone in the medullary cavity at the fracture ends, and the medullary cavity was not reconnected (Fig. 4C).

### Micro-CT evaluation of fracture healing and callus formation

**Figure 5.**
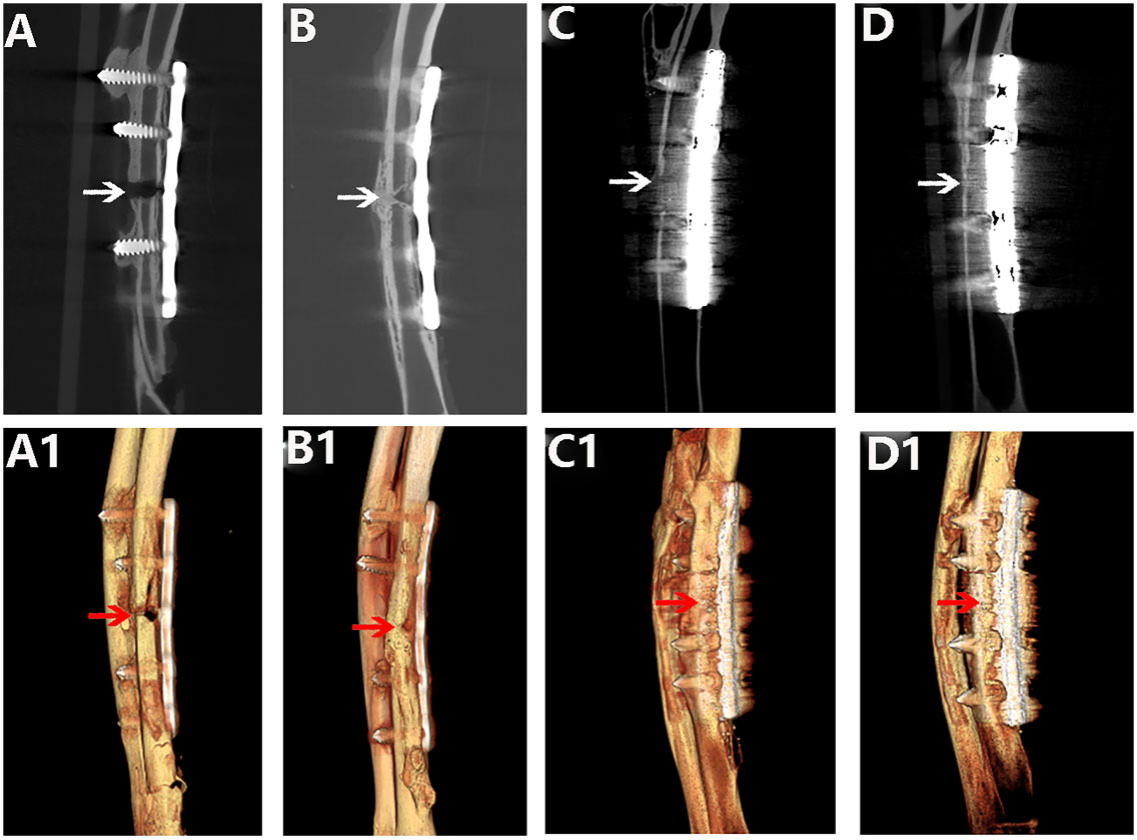
Micro-CT assessment of the healing process of rabbit ulnar fractures in the Ti plate group and pTa trabecular bone metal plate group: (**A** and **A1**) CT images of the Ti plate group 4 weeks after surgery: the fracture line is clear, and there is no new callus formation around the fracture; (**B** and **B1**) CT image of the Ti plate group 12 weeks after surgery. It can be seen that the fracture has healed and there is callus formation around the fracture line. (**C** and **C1**) CT images of the pTa plate group at 4 weeks postoperatively: the fracture line is blurred, and there is callus formation around the fracture line and the bone plate; (**D** and **D1**) CT images of the pTa plate group at 12 weeks postoperatively, fracture healed, a large number of callus formed around the fracture line and bone plate. *Note*: the arrow represents the fracture gap.

The state of rabbit ulnar fracture healing and callus tissue formation was observed and evaluated by micro-CT. Fracture fixation was achieved with both the pTa trabecular metal plates and Ti plates, without reduction or breakage of bone mass. No osteonecrosis was found below the plates in either group, but at 12-week, sclerotic bone was found at the fracture ends in the Ti group, the medullary cavity was still not connected, and osteoporosis was found below the plate (Fig. 5B). In the pTa trabecular metal plate group, extensive callus tissue was found on the coronal plane by micro-CT in the fracture gap and around the plate (Fig. 5C1). Micro-CT at Week 12 (Fig. 5D1) showed that the bone at the fracture gap had been well reconstructed into relatively normal bone in terms of appearance and shape; additionally, the medullary cavity was reconnected, and there was no osteoporosis under the plate. Meanwhile, in the process of fracture healing, the new callus wrapped the fracture and the pTa trabecular metal plate closely. Over time, the osteoid callus tissue gradually became hard callus tissue, and the plate wrapped by the new bone tissue became a part of the bone, increasing the strength of the bone after fracture healing.

### Histological evaluation of fracture healing and the bone–plate interface

**Figure 6.**
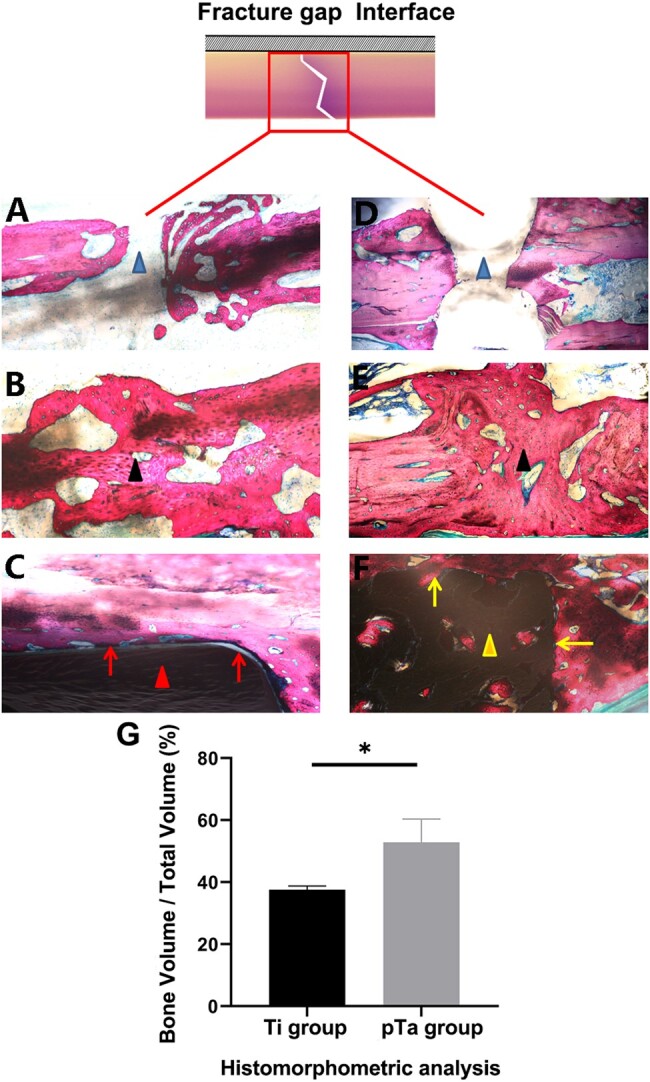
Van Gieson staining to assess callus formation and the interface of the bone and plate at 4 and 12 weeks in the Ti (**A–C**) and pTa (**D–F**) plate groups. (A and D) Fracture gap (at ×40 magnification); (B and E) healing status at the fracture gap (at ×100 magnification); (C and F) interface between the pTa or Ti plate and bone (at ×100 magnification). (G) Histomorphometric analysis of the Ti and pTa groups at 12 weeks after surgery. Asterisks (*) indicate statistical significance compared to the Ti group, *P* < 0.05. *Note*: the blue triangle represents the fracture gap; the black triangle represents the new bone tissue; the red triangle represents the Ti plate; the yellow triangle represents the pTa plate; the red arrow represents the bone– Ti plate interface; the yellow arrow represents the bone–pTa plate interface; the red box represents the fracture gap.

Specimens of fracture healing at 4 and 12 weeks were evaluated for healing and osseointegration of the metal by histological analysis based on Van Gieson staining. The results showed that at 4-week after the operation, the fracture lines in the Ti group (Fig. 6A) and the pTa trabecular metal plate group (Fig. 6D) were obvious, and no new bone tissue had formed in the fracture gap or around the fracture. At Week 12 in the Ti group (Fig. 6B), the trabecular bone in the fracture gap had become thicker and denser, and the trabecular bone gap had narrowed, but no mature lamellar bone had formed. There were gaps in almost all regions of the interface between the Ti plate and the bone tissue (Fig. 6C), which indicated that new bone would not grow on the Ti surface. At Week 12, the bone tissue at the fracture in the pTa trabecular metal plate group (Fig. 6E) had changed shape; the fracture gap had disappeared and been filled with coarse trabecular bone, and these trabecular structures had almost become mature lamellar bone. At the same time, we observed the formation of mature bone tissue in the porous structure of the pTa trabecular metal plate and the interface between the plate and bone. The new bone tissue was tightly attached to the pTa trabecular metal plate and the pores, without gaps (Fig. 6F).

No degradation was observed in either group during healing. Additionally, no bone resorption, osteonecrosis, or inflammation was observed below the plate at any time point by histological observation.

## Discussion

The main goal of treating fractures is to rapidly rehabilitate the bone and restore limb function. The Association for the Study of Internal Fixation (ASIF) has promoted the core idea of strong fracture fixation to maintain absolute stability of the fracture ends and pursue direct fracture healing [19]. Metal plates have become the first choice for internal fixation due to their high strength [20]. However, the elastic modulus of metal plates is tens of times that of cortical bone. After implantation, these plates participate in load transfer, resulting in stress shielding that is not conducive to fracture healing [21, 22]. Until the 1990s, ASIF scholars proposed biological bone grafting technology, with the idea that ‘fracture treatment must focus on seeking a balance between fracture stability and soft tissue integrity’ [23]. They did not seek rigid fracture fixation but rather elastic fracture fixation with the protection of soft tissue and preservation of the fracture end blood supply. Although this treatment concept has made more progress than the ASIF concept, it only changed the fixation method and did not propose changes to the nature of the material. To overcome these shortcomings of metal materials, degradable materials have attracted the interest of scientists and orthopedic surgeons for some time, and degradable polylactic acid- and magnesium-based metals have been evaluated as materials for internal fixation devices. These materials have low elastic moduli, stable performance and no interference with radiation-based imaging [9]. However, the fixation strength and side effects after the degradation of these degradable materials are still problems that limit their clinical application [24].

The objective of this study was to manufacture a novel plate material. The bone plates have good mechanical strength and a low elastic modulus and can effectively fix fractures without stress shielding, which also have good biological activity to promote fracture healing and can be retained in the body for a long time without reoperation. Therefore, this study used CVD technology to prepare a novel pTa trabecular metal plate by coating porous SiC scaffolds with Ta metal. Earlier research results have shown that the novel pTa trabecular metal has excellent biocompatibility and biomechanical properties and thus potential as an internal fixation device [16–18]. So, this study prepared a new type of pTa trabecular metal plate and further evaluated the effect of the novel plate as an internal fixation device through an *in vivo* implantation experiment.

In the course of this study, through frequent imaging, we observed that the metal plates in both groups were firmly fixed the rabbit ulnar fractures without breakage, demonstrating the good strength and mechanical properties of both plates, which are the primary requirements for an internal fixation plate. At present, the most common internal fixation system used in the clinical treatment of fractures is the Ti locking plate system. In the process of implantation, Ti locking plate fixation away from the bone surface is the preferred method [25], mainly because this approach can prevent interference with the reparability of the surface periosteum and maintain adequate blood flow; however, its stress shielding has not been overcome [26]. The elastic modulus of pTa trabecular metal plates is 4.8 GPa, which is much lower than that of Ti metal plates [27, 28]. The Young’s modulus of pTa trabecular metal plates is similar to that of cortical bone; thus, the pTa trabecular metal plate used in this study did not cause stress shielding and can be implanted to enhance the strength of bone during fracture healing. In addition, during the operation, pTa trabecular metal plates are easier to shape according to the anatomical morphology of the bone than Ti plates, and it exhibits better adhesion to bone. In the process of fixation, pTa trabecular metal plates cannot achieve the rigid fixation of Ti locking plates, but their performance is in line with the concept of elastic biological fixation, especially in the repair of comminuted fractures involving small bone fragments. While allowing the micromotion between fracture fragments to stimulate callus formation, pTa trabecular metal plates also ensure the stable fixation of small bone fragments and provides a better environment for fracture healing.

Through frequent imaging of the process of fracture healing, in the pTa trabecular metal plates group, a large number of callus formation of the fracture group was observed which surrounded even the bone plate. The Ti plate group had relatively few calluses formation. This also proves that the method of fracture fixation of Ti plates is rigid fixation, and the method of fixing the fracture of the pTa trabecular metal plates is elastic fixation, and the fracture is healed by indirect healing method. The Micro-CT confirms that the new callus is closely wrapped around the pTa trabecular metal plates, and the pTa trabecular metal plates are integrated with the host bone, which further enhances the strength of the bone after the fracture healing. Previous preclinical and clinical experience supported the callus formation of the fracture in the process of indirect healing which further supports the new bone [29], reducing the risk of re-fracture. This also indirectly proves that although the pTa trabecular metal plates do not have a high bending strength of the Ti plates, they will not break the plate when it is used to withstand a fracture of large loads alone [7].

At 12-week after the operation, imaging showed that the fractures in both groups had healed completely, and the medullary cavity in the pTa trabecular metal plate group was completely reconnected. However, in the Ti plate group, there was still sclerotic bone in the medullary cavity at the fracture, and osteoporosis was found below the plate. This was mainly because of the rigid fixation provided by the Ti plate; after fracture healing, the stiffness or elastic modulus of the bone and plate does not match, eventually leading to bone mass reduction and even bone embrittlement, reducing the initial strength of fracture healing [30]. Therefore, ideal internal fixation should achieve a balance between biological and mechanical stability and should conform to the concept of biological fixation. The novel pTa trabecular metal plate provided elastic fixation, which can promote callus formation and fracture healing. A low elastic modulus has no adverse impact on bone loss in the process of fracture healing and is suitable for long-term bone implants.

The interface between bone and an implant affects the long-term effect of internal fixation devices. A good interface is conducive to implant stability and reducing the shear force at the bone–plate interface. Because the pTa trabecular metal plate is a porous structure, it can better retain cells; when implanted *in vivo*, the surrounding osteoblasts can quickly adhere to its surface and grow into pores, transitioning mechanical-only fixation to mechanical–biological fixation. At Week 12 after implantation, the histological results showed that the new callus surrounded the host bone and pTa trabecular metal plate; extensive new bone had formed both around the plate and in the pores of the plate, demonstrating the good biocompatibility of the plate with the host bone. At this stage, the pTa trabecular metal plate was integrated with the new bone to become a part of the host bone, which further enhanced the strength of the bone after fracture healing. The stress was evenly distributed in the pTa trabecular metal plate and the host bone after fracture healing to prevent stress shielding and help to achieve long-term support. Finally, the plate was integrated with the host bone to achieve the effect of biological fixation without the need for reoperation. Therefore, the implantation of pTa trabecular metal plates for long-term retention *in vivo* is safe.

Besides having excellent biocompatibility and bone integration performance, the pTa trabecular metal plates can also promote bone repair and reconstruction. However, CT image shows that the pTa trabecular metal plates had a stronger metal pseudo shadow than the Ti plates. The presence of metal pseudo shadows can affect the imaging effect, which may obstruct the clearer observation of the fracture healing process. Although the parameters and scanning modes of the detection device can be adjusted to reduce the metal pseudo shadows, complete removal is not possible due to high magnetization rate of the pTa trabecular metal. Therefore, in subsequent studies, it is needed to focus on surface structural research or surface coating modification of the pTa trabecular metal to reduce the magnetization rate of pTa trabecular metal products.

## Conclusions

In this study, we have demonstrated that pTa trabecular bone metal plates can be used to fix fractures without causing breakage or stress shielding. Unlike traditional metal plates, these novel pTa trabecular bone metal plates can promote fracture healing and integrate well with host bone without adverse effects on the bone tissue; thus, they can be retained *in vivo* for a long time. These research results have proven the feasibility and advantages of trabecular bone metal plates in the internal fixation of fractures.
